# Supplementary material for: Infrared spectroscopic study of hydrogen bonding topologies in the smallest ice cube
Source: Nat Commun. 2020 Oct 28;11:5449. doi: 10.1038/s41467-020-19226-6 (PMC7595032; doi:10.1038/s41467-020-19226-6)
Supplement: Supplementary file 1 — Supplementary Information [file 41467_2020_19226_MOESM1_ESM.pdf]

Supplementary Information

Infrared spectroscopic study of hydrogen bonding topologies in the smallest ice cube

Gang Li[1,5], Yang-Yang Zhang[2,5], Qinming Li[1,3,5], Chong Wang[1,3], Yong Yu[1,3], Bingbing Zhang[1], Han-Shi Hu[2], Weiqing Zhang[1], Dongxu Dai[1], Guorong Wu[1], Dong H. Zhang[1], Jun Li[2,4]*, Xueming Yang[1,4]*, & Ling Jiang[1]*

[1]State Key Laboratory of Molecular Reaction Dynamics, Dalian Institute of Chemical Physics, Chinese Academy of Sciences, Dalian 116023, China.

[2]Key Laboratory of Organic Optoelectronics & Molecular Engineering of the Ministry of Education, Department of Chemistry, Tsinghua University, Beijing 100084, China.

[3]University of Chinese Academy of Sciences, 19A Yuquan Road, Beijing 100049, China.

[4]Department of Chemistry, Southern University of Science and Technology, Shenzhen 518055, China.

[5]These authors contributed equally: Gang Li, Yang-Yang Zhang, Qinming Li.

*email: junli@mail.tsinghua.edu.cn; xmyang@dicp.ac.cn; ljiang@dicp.ac.cn.

This PDF file includes:

S1. Supplementary Figures 1 to 8 page 2-9

S2. Supplementary Tables 1 to 16 page 10-29

S3. Supplementary References page 30

S1. Supplementary Figures

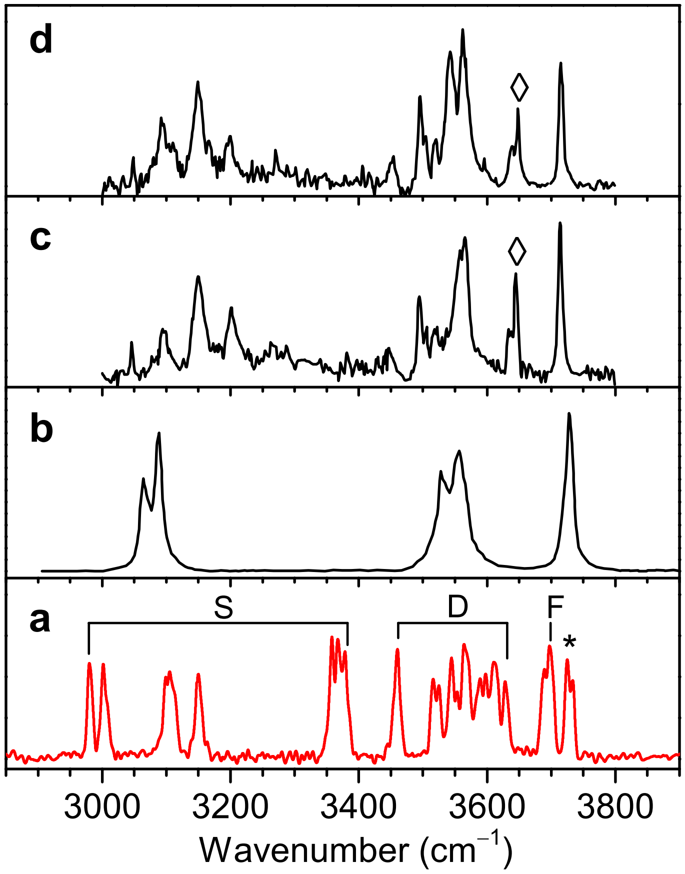

Supplementary Fig. 1 Comparison of present (a) and previously (b–d) measured IR spectra of $(H_2O)_8$. a IR-VUV spectroscopy based on threshold photoionization using a tunable vacuum ultraviolet free electron laser (this work). **b** Helium-scattering IR spectroscopy based on electron impact ionization[1]. IR-UV spectra of benzene-$(H_2O)_8$ measured at the transitions 70.3 cm^{-1} (benzene-$(H_2O)_8$, D_{2d}) of **c** and 67.6 cm^{-1} (benzene-$(H_2O)_8$, S_4) above the 6_0^1 transition of free benzene of **d**[2]. The band labeled with "◊" is due to the π hydrogen-bonded OH stretch[2].

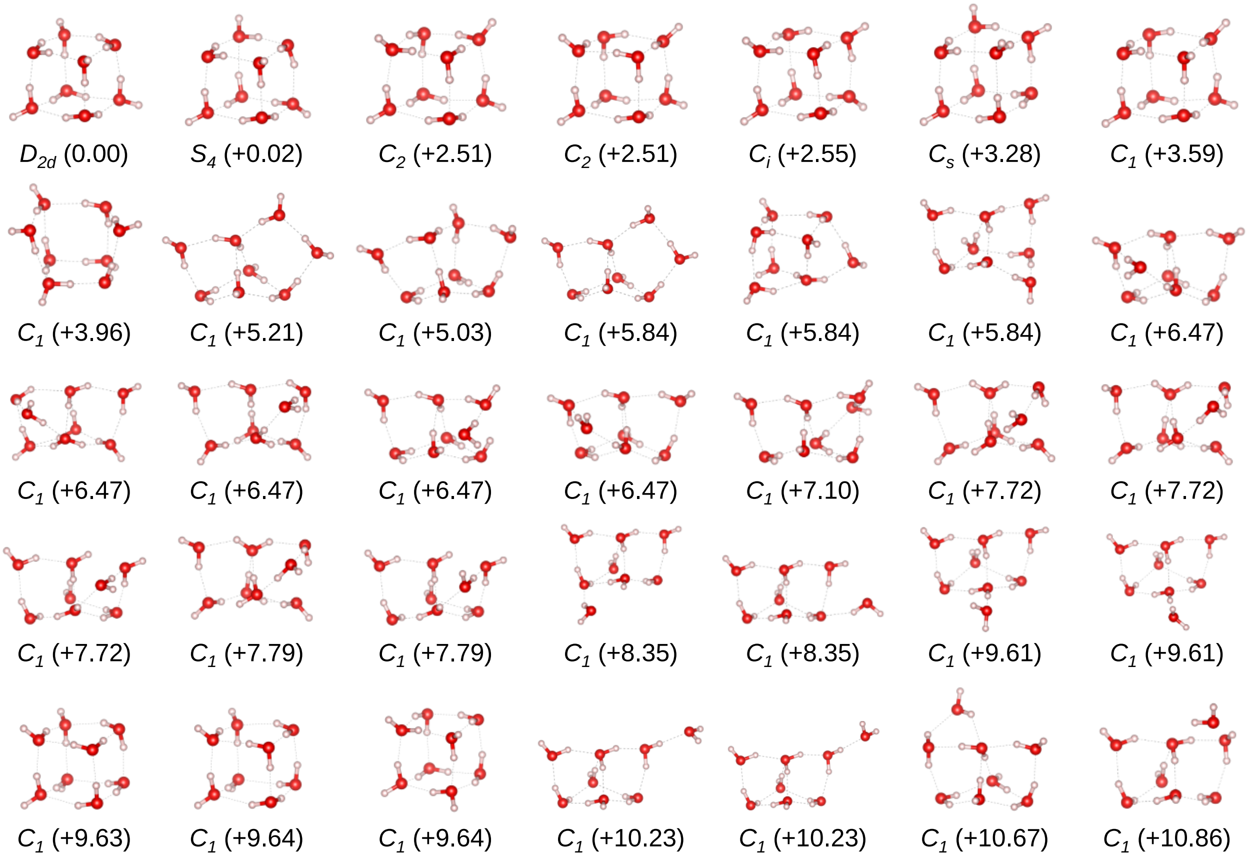

Supplementary Fig. 2 Low-lying structures of $(H_2O)_8$ obtained from global minimum search by TGMin (O, red; H, light gray). MP2/AVDZ relative energies (in kcal/mol) are listed inside the parenthesis.

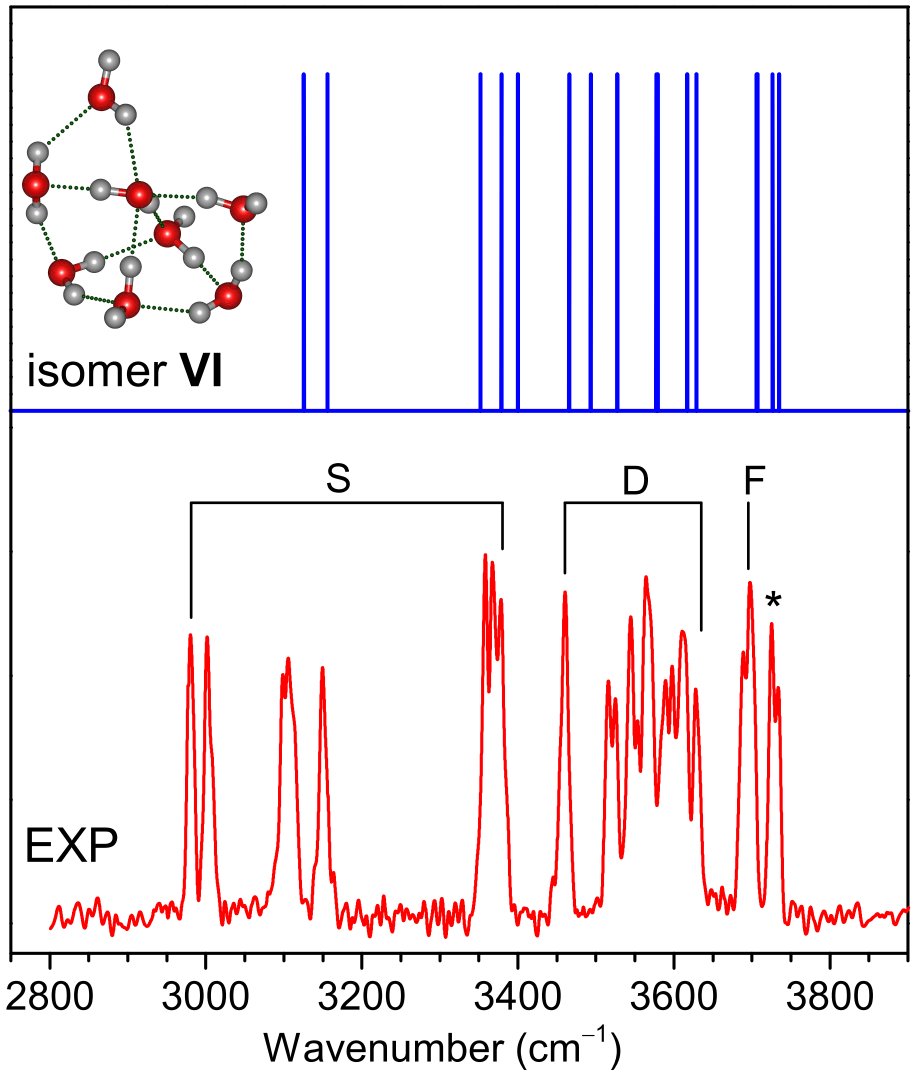

Supplementary Fig. 3 Comparison of the experimental IR spectrum of $(H_2O)_8$ with the calculated IR spectrum of isomer VI. The OH stretch fundamentals assigned to H-donor-free OH (F), double H-donor OH stretch (D), and single H-donor OH stretch (S) are labeled. The calculations were performed at the MP2/AVDZ level, with the harmonic frequencies scaled by 0.956. The structure of isomer **VI** is embedded in the inset (O, red; H, light gray). Isomer **VI** consists of a meta-stable one-water-solvated heptamer, which yields calculated positions of donor-free OH stretches at 3706 and 3735 cm^{-1} with a separation of 29 cm^{-1}, consistent with the experimental band positions (3698 and 3726 cm^{-1}) and splitting (30 cm^{-1}).

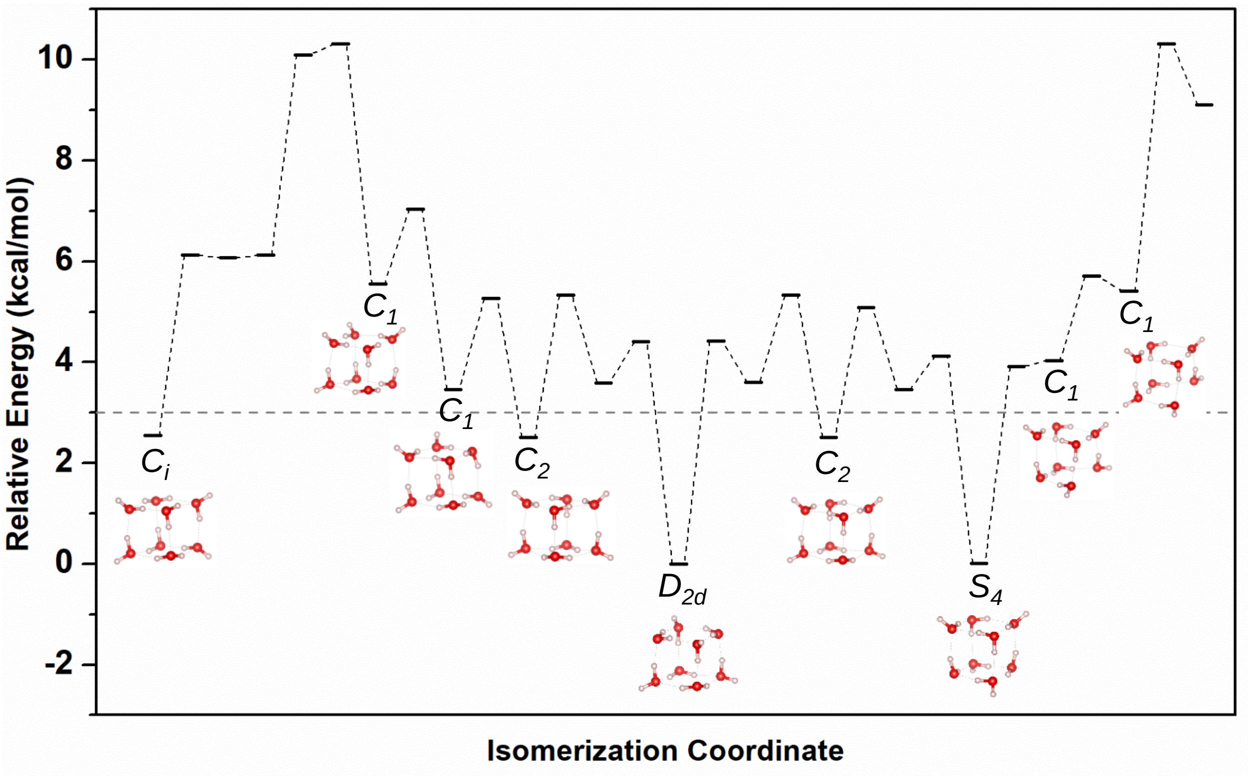

Supplementary Fig. 4 The energy profile showing transition states between different isomers of $(H_2O)_8$ (O, red; H, light gray).

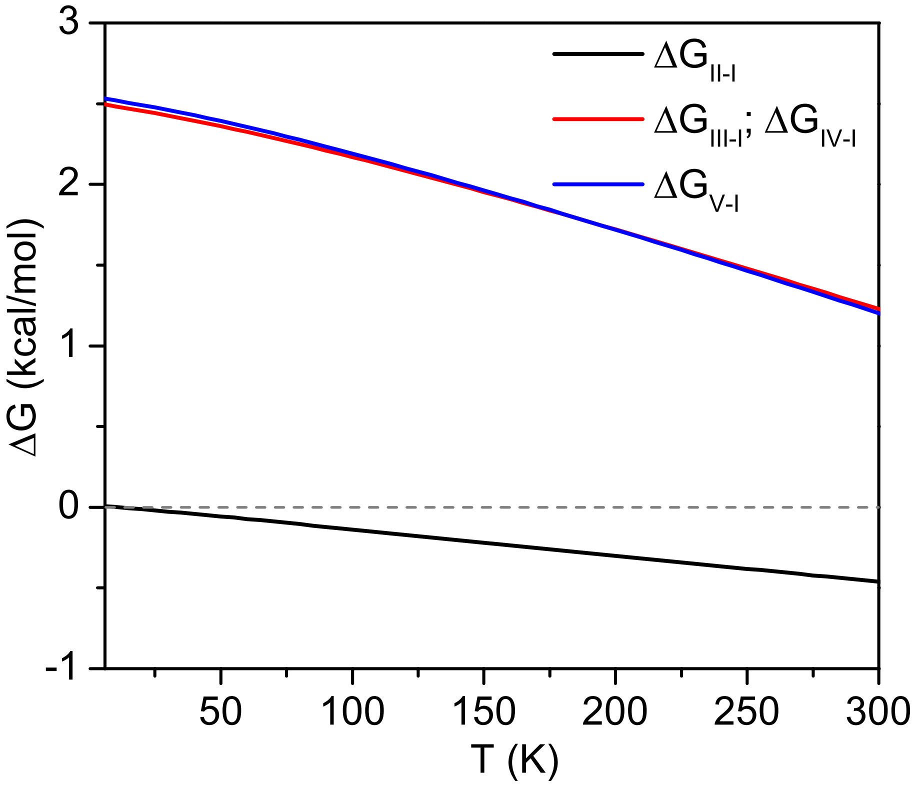

Supplementary Fig. 5 Gibbs free energies of isomers II−V relative to isomer I as a function of temperature. The calculations were carried out at the MP2/AVDZ level.

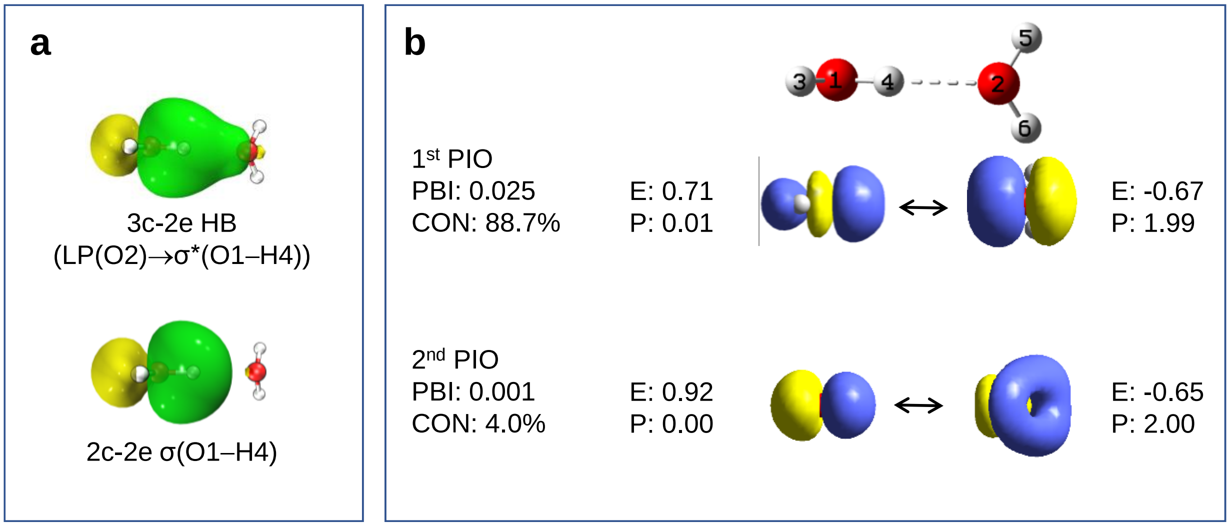

Supplementary Fig. 6 a AdNDP bonding analyses for $(H_2O)_2$ at the MP2/AVDZ level of theory (O, red; H, light gray). Hydrogen bond is abbreviated as HB, and lone pair as LP. Occupation numbers (ON) are 2.00 |e|. **b** Results of PIO analysis on $(H_2O)_2$ with two H_2O molecules as two fragments (O, red; H, light gray). The first five PIOs of each fragment are shown here. PIO-based bond indices are abbreviated as PBI, the contribution to the total interactions between two fragments as (CON), the orbital energies as E, and populations (occupation numbers) as P. Isovalue of orbital is 0.02.

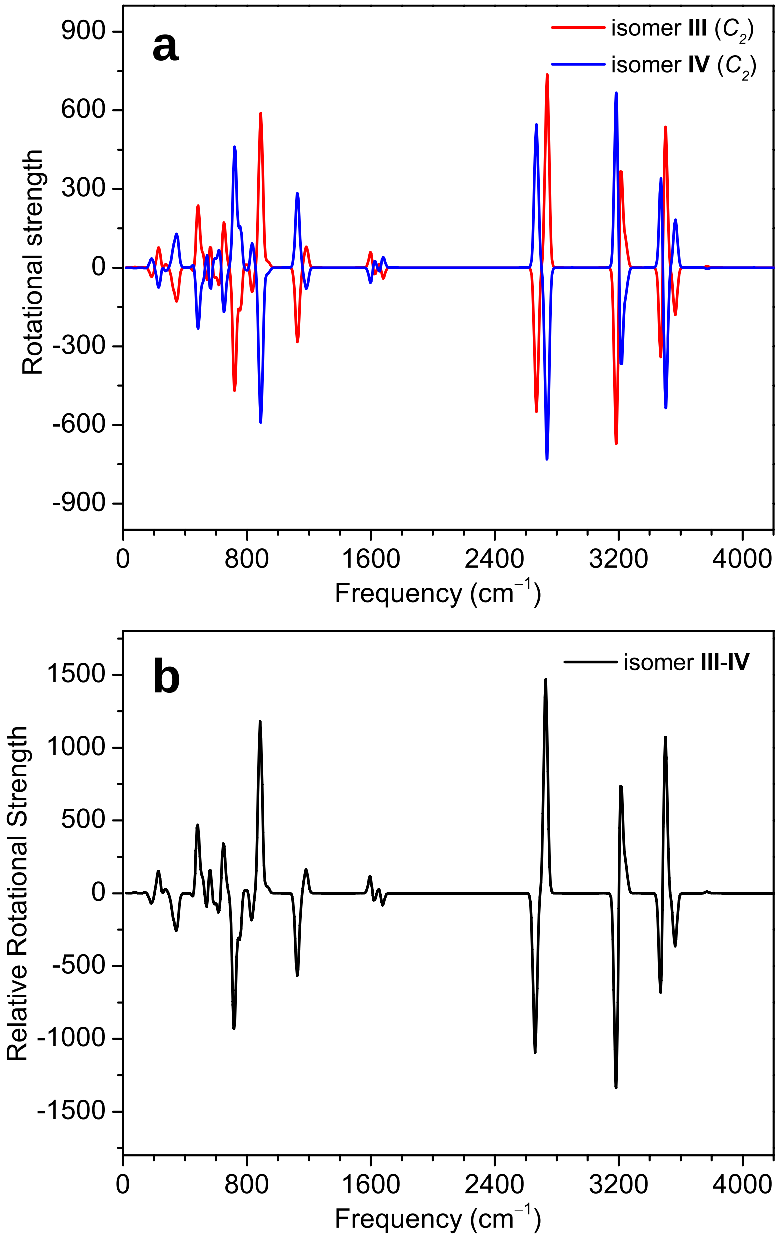

Supplementary Fig. 7 The vibrational circular dichroism (VCD) spectra of two chiral water octamers (isomers III and IV) calculated at the PBE/TZ2P level of theory. a Rotational strength and **b** the relative rotational stretch of isomers **III** and **IV** as a function of frequency.

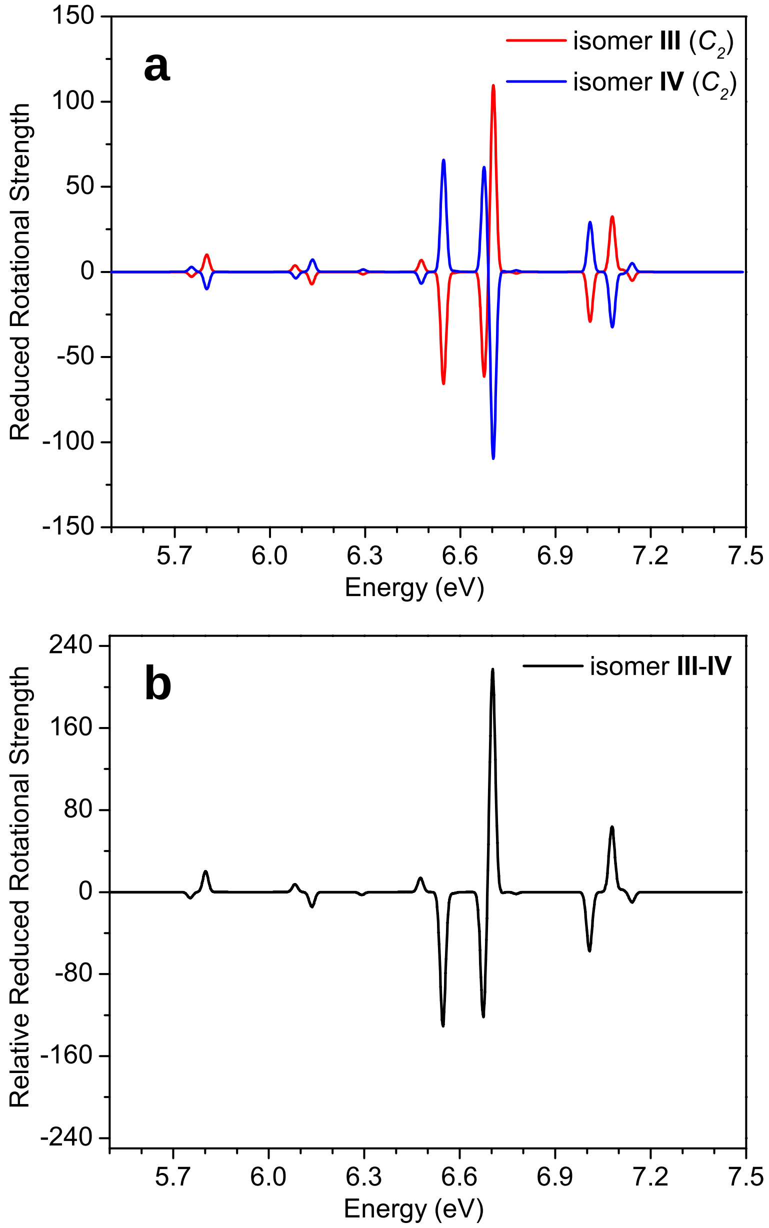

Supplementary Fig. 8 The electronic circular dichroism (ECD) spectra of two chiral water octamers (isomers III and IV) calculated at the PBE/TZ2P level of theory. a Reduced rotational strength and **b** the relative reduced rotational stretch of isomers III and IV as a function of energy.

S2. Supplementary Tables

Supplementary Table 1. Experimental vibrational frequencies, widths, and band assignments for $(H_2O)_8$. The widths are full widths at half-maximum (FWHM).

Label	Frequency (cm^{-1})	FWHM width (cm^{-1})	Assignment
S	2980	9	single H-donor OH stretch
	3002	12	
	3106	20	
	3150	10	
	3378	43	
D	3460	12	double H-donor symmetric OH stretch (D^{sym})
	3516	18	
	3526–3628	–	double H-donor antisymmetric OH stretch (D^{asym})
F	3698	24	H-donor-free OH
*	3726	18	H-donor-free OH

Supplementary Table 2. Scaled harmonic vibrational frequencies in the OH stretching region of isomer **I** (D_{2d}) of $(H_2O)_8$ calculated at the MP2/AVDZ level of theory (O, red; H, light gray).

mode numbers	frequencies (cm^{-1})	intensities (km/mol)	assignment	displacement vectors
ν_1	3083	0	S	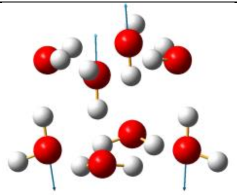
ν_2	3107	17	S	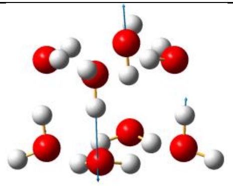
ν_3	3107	17	S	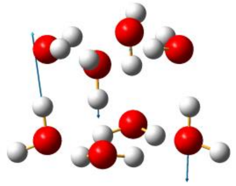
ν_4	3164	3288	S	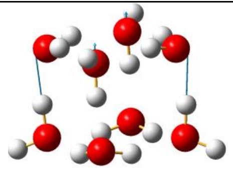
ν_5	3443	386	D^{sym}	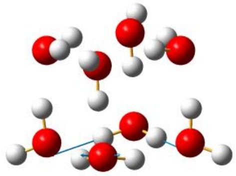
ν_6	3443	386	D^{sym}	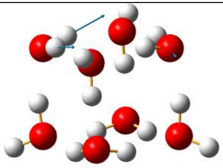
ν_7	3459	0	D^{sym}	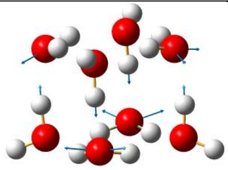

ν_8	3461	21	D^{sym}	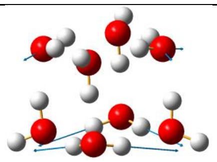
ν_9	3506	0	D^{asym}	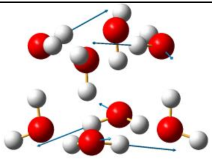
ν_{10}	3507	0	D^{asym}	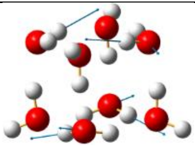
ν_{11}	3551	1087	D^{asym}	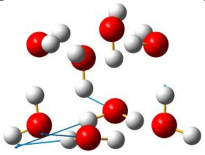
ν_{12}	3551	1087	D^{asym}	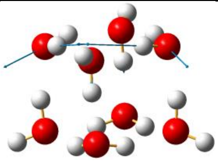
ν_{13}	3708	70	F	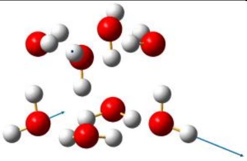
ν_{14}	3708	70	F	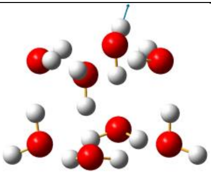
ν_{15}	3708	175	F	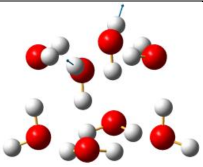
ν_{16}	3708	0	F	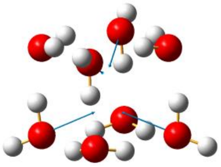

Supplementary Table 3. Scaled harmonic vibrational frequencies in the OH stretching region of isomer **II** (*S_4*) of $(H_2O)_8$ calculated at the MP2/AVDZ level of theory (O, red; H, light gray).

mode numbers	frequencies (cm^{-1})	intensities (km/mol)	assignment	displacement vectors
ν_1	3080	0	S	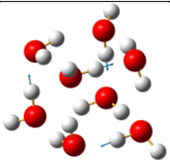
ν_2	3104	19	S	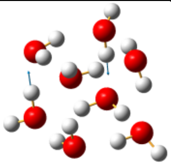
ν_3	3131	1622	S	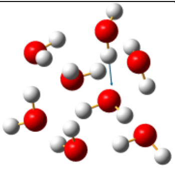
ν_4	3131	1622	S	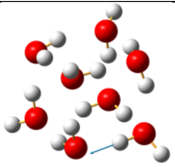
ν_5	3444	418	D^{sym}	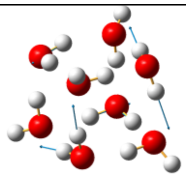
ν_6	3450	165	D^{sym}	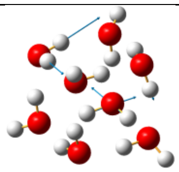
ν_7	3450	165	D^{sym}	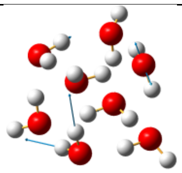
ν_8	3460	0	D^{sym}	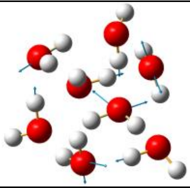

ν_9	3505	0	D^{asym}	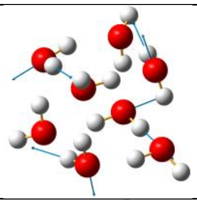
ν_{10}	3531	628	D^{asym}	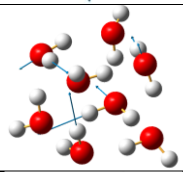
ν_{11}	3531	628	D^{asym}	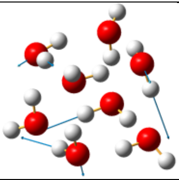
ν_{12}	3548	1081	D^{asym}	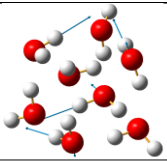
ν_{13}	3708	69	F	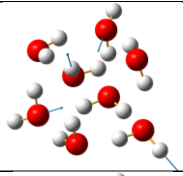
ν_{14}	3708	123	F	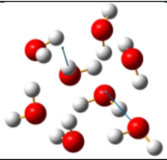
ν_{15}	3708	123	F	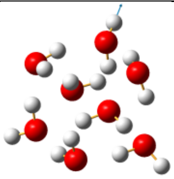
ν_{16}	3708	0	F	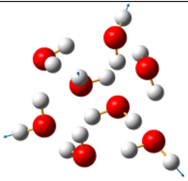

Supplementary Table 4. Scaled harmonic vibrational frequencies in the OH stretching region of isomers **III** and **IV** (C_2) of $(H_2O)_8$ calculated at the MP2/AVDZ level of theory (O, red; H, light gray).

mode numbers	isomer III (C_2)		isomer IV (C_2)		assign-ment	displacement vectors
	Freq. (cm^{-1})	intensities (km/mol)	Freq. (cm^{-1})	intensities (km/mol)		
ν_1	2964	7	2964	7	S	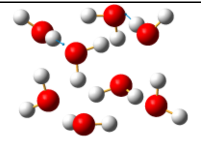
ν_2	3006	1993	3006	1993	S	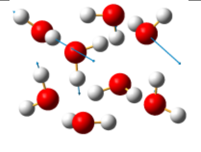
ν_3	3312	16	3312	16	S	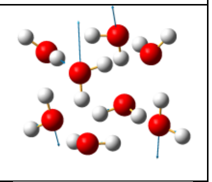
ν_4	3316	27	3316	27	S	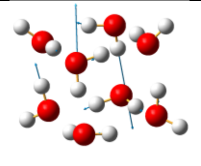
ν_5	3328	43	3328	43	S	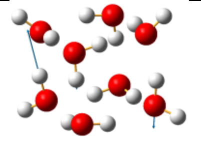
ν_6	3348	2021	3348	2021	S	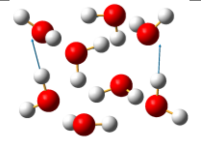
ν_7	3487	243	3487	243	D^{sym}	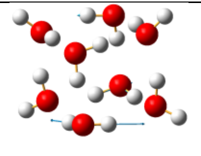
ν_8	3500	22	3500	22	D^{sym}	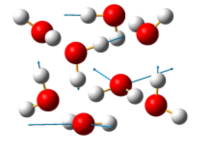

ν_9	3536	149	3536	149	D^{asym}	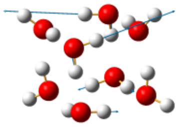
ν_{10}	3541	651	3541	651	D^{asym}	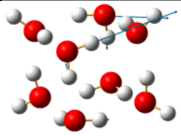
ν_{11}	3564	0	3564	0	D^{asym}	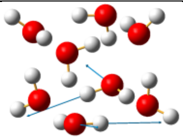
ν_{12}	3599	892	3599	892	D^{asym}	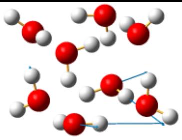
ν_{13}	3702	71	3702	71	F	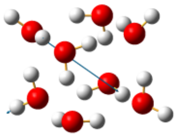
ν_{14}	3702	82	3702	82	F	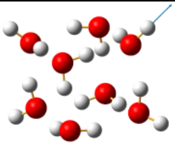
ν_{15}	3711	79	3711	79	F	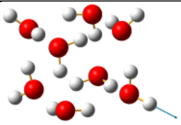
ν_{16}	3711	111	3711	111	F	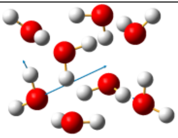

Supplementary Table 5. Scaled harmonic vibrational frequencies in the OH stretching region of isomer **V** (C_i) of $(H_2O)_8$ calculated at the MP2/AVDZ level of theory (O, red; H, light gray).

mode numbers	frequencies (cm^{-1})	intensities (km/mol)	assignment	displacement vectors
ν_1	2971	1	S	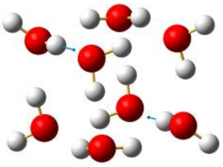
ν_2	2994	2041	S	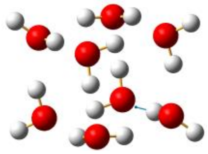
ν_3	3311	47	S	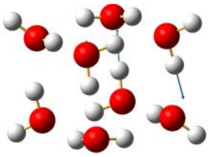
ν_4	3318	0	S	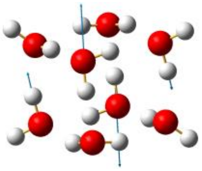
ν_5	3326	0	S	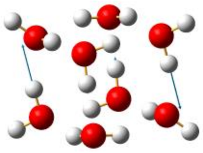
ν_6	3346	2055	S	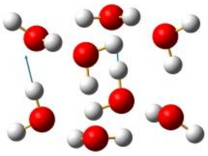
ν_7	3490	0	D^{sym}	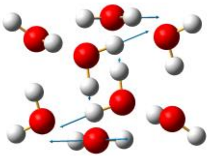
ν_8	3496	291	D^{sym}	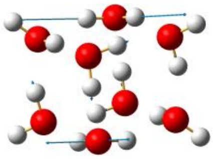

ν_9	3535	6	D^{asym}	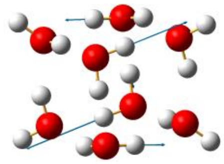
ν_{10}	3535	511	D^{asym}	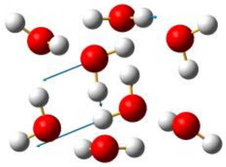
ν_{11}	3581	0	D^{asym}	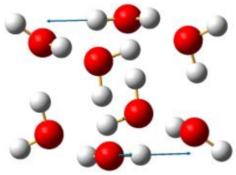
ν_{12}	3587	1135	D^{asym}	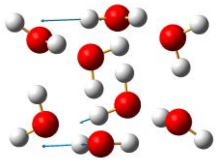
ν_{13}	3702	132	F	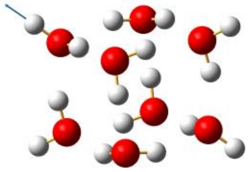
ν_{14}	3702	15	F	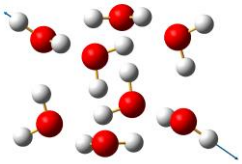
ν_{15}	3711	10	F	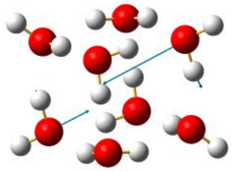
ν_{16}	3711	176	F	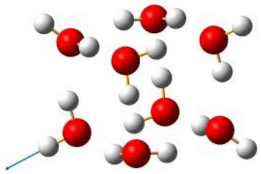

Supplementary Table 6. The percentage of the contribution of hydrogen bond energy to the total bond energy (E_{HB}/E_{total}) of cubic isomers **I–V** for the water octamer of theory (O, red; H, light gray).

isomer	E_{HB}/E_{total} (%)
**I**	89.01
**II**	89.00
**III**	89.00
**IV**	89.33
**V**	89.31

Supplementary Table 7. The O-O, O–H, and H···O distances (in Å) of isomer **I** (D_{2d}) for $(H_2O)_8$ calculated at the MP2/AVDZ level of theory (O, red; H, light gray). The second order perturbation energies (E_2, in kcal/mol) were obtained from Natural Bond Orbital (NBO) calculations at the same theory level.

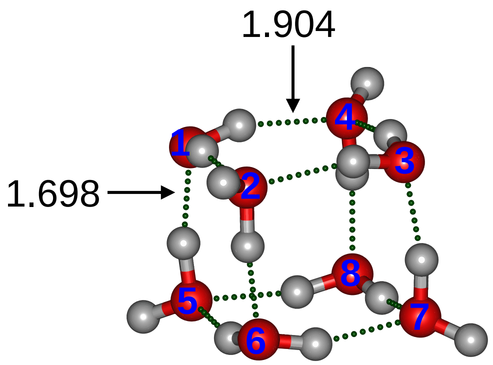

	d(O-O)	d(O–H)	d(H···O)	E_2
O^1-O^5	2.694	0.996	1.698	32.17
O^2-O^6	2.694	0.996	1.698	32.17
O^3-O^7	2.694	0.996	1.698	32.17
O^4-O^8	2.694	0.996	1.698	32.17
O^1-O^2	2.881	0.977	1.904	12.64
O^2-O^3	2.881	0.977	1.904	12.64
O^3-O^4	2.881	0.977	1.904	12.64
O^4-O^1	2.881	0.977	1.904	12.64
O^5-O^6	2.881	0.977	1.904	12.64
O^6-O^7	2.881	0.977	1.904	12.64
O^7-O^8	2.881	0.977	1.904	12.64
O^8-O^5	2.881	0.977	1.904	12.64

Supplementary Table 8. The O-O, O–H, and H···O distances (in Å) of isomer **II** (S_4) for $(H_2O)_8$ calculated at the MP2/AVDZ level of theory (O, red; H, light gray). The second order perturbation energies (E_2, in kcal/mol) were obtained from Natural Bond Orbital (NBO) calculations at the same theory level.

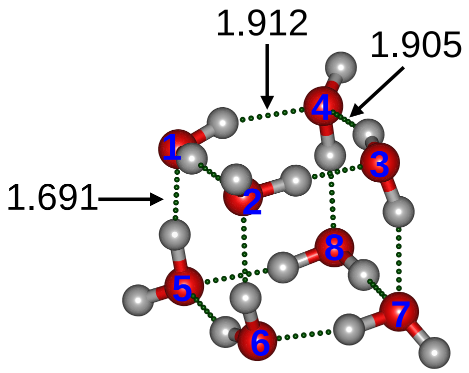

	d(O-O)	d(O–H)	d(H···O)	E_2
O^1-O^5	2.672	0.996	1.691	32.19
O^2-O^6	2.852	0.977	1.912	7.91
O^3-O^7	2.852	0.977	1.912	7.91
O^4-O^8	2.672	0.996	1.691	32.19
O^1-O^2	2.849	0.977	1.905	9.06
O^2-O^3	2.672	0.996	1.691	32.19
O^3-O^4	2.849	0.977	1.905	9.06
O^4-O^1	2.852	0.977	1.912	7.91
O^5-O^6	2.849	0.977	1.905	9.06
O^6-O^7	2.672	0.996	1.691	32.19
O^7-O^8	2.849	0.977	1.905	9.06
O^8-O^5	2.852	0.977	1.912	7.91

Supplementary Table 9. The O-O, O–H, and H···O distances (in Å) of isomer **III** (*C_2*) for $(H_2O)_8$ calculated at the MP2/AVDZ level of theory (O, red; H, light gray). The second order perturbation energies (E_2, in kcal/mol) were obtained from Natural Bond Orbital (NBO) calculations at the same theory level.

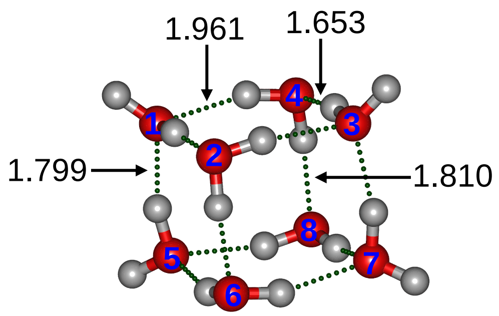

	d(O-O)	d(O–H)	d(H···O)	E_2
O^1-O^5	2.754	0.984	1.799	19.43
O^2-O^6	2.774	0.985	1.810	21.00
O^3-O^7	2.754	0.984	1.798	19.43
O^4-O^8	2.754	0.984	1.799	19.43
O^1-O^2	2.644	1.003	1.653	37.95
O^2-O^3	2.890	0.975	1.961	10.05
O^3-O^4	2.644	1.003	1.653	37.95
O^4-O^1	2.890	0.975	1.961	10.05
O^5-O^6	2.903	0.975	1.965	5.39
O^6-O^7	2.886	0.975	1.945	7.10
O^7-O^8	2.903	0.975	1.965	5.39
O^8-O^5	2.886	0.975	1.945	7.10

Supplementary Table 10. The O-O, O–H, and H···O distances (in Å) of isomer **IV** (C_2) for $(H_2O)_8$ calculated at the MP2/AVDZ level of theory (O, red; H, light gray). The second order perturbation energies (E_2, in kcal/mol) were obtained from Natural Bond Orbital (NBO) calculations at the same theory level.

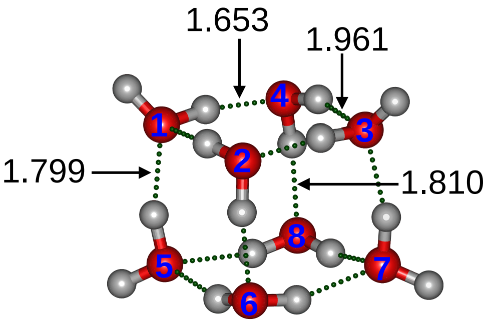

	d(O-O)	d(O–H)	d(H···O)	E_2
O^1-O^5	2.754	0.984	1.799	19.43
O^2-O^6	2.774	0.985	1.810	21.00
O^3-O^7	2.754	0.984	1.799	19.43
O^4-O^8	2.754	0.984	1.799	19.43
O^1-O^2	2.890	0.975	1.961	10.05
O^2-O^3	2.644	1.003	1.653	37.95
O^3-O^4	2.890	0.975	1.961	10.05
O^4-O^1	2.644	1.003	1.653	37.95
O^5-O^6	2.886	0.975	1.945	7.10
O^6-O^7	2.903	0.975	1.965	5.39
O^7-O^8	2.886	0.975	1.945	7.10
O^8-O^5	2.903	0.975	1.965	5.39

Supplementary Table 11. The O-O, O–H, and H···O distances (in Å) of isomer V (C_i) for $(H_2O)_8$ calculated at the MP2/AVDZ level of theory (O, red; H, light gray). The second order perturbation energies (E_2, in kcal/mol) were obtained from Natural Bond Orbital (NBO) calculations at the same theory level.

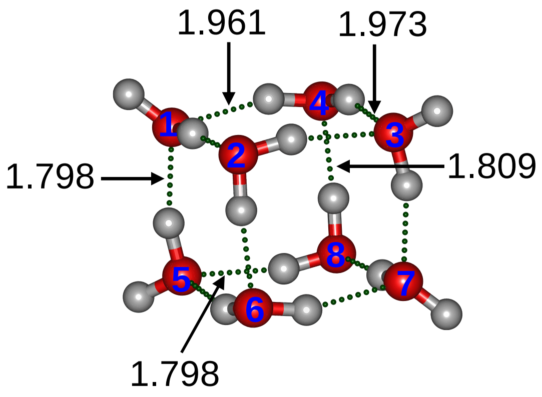

	d(O-O)	d(O–H)	d(H···O)	E_2
O^1-O^5	2.754	0.985	1.798	19.47
O^2-O^6	2.773	0.986	1.808	21.02
O^3-O^7	2.754	0.985	1.798	19.44
O^4-O^8	2.774	0.986	1.809	20.97
O^1-O^2	2.640	1.003	1.648	38.37
O^2-O^3	2.883	0.975	1.948	8.14
O^3-O^4	2.910	0.974	1.973	6.27
O^4-O^1	2.898	0.975	1.961	10.08
O^5-O^6	2.910	0.974	1.973	6.29
O^6-O^7	2.898	0.975	1.961	10.08
O^7-O^8	2.641	1.003	1.649	38.32
O^8-O^5	2.883	0.975	1.948	8.17

Supplementary Table 12. Wiberg bond order and natural hybrid orbital of the single H-donor OH, double H-donor OH, and H-donor-free OH bonds in isomer **I** (D_{2d}) for $(H_2O)_8$ calculated at the MP2/AVDZ level of theory (O, red; H, light gray).

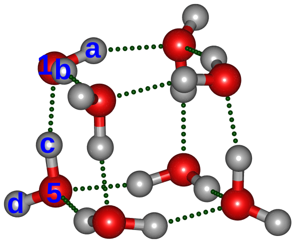

Type of OH group	Bond	Wiberg bond order	Natural hybrid orbital
single H-donor OH	O^5–H^c	0.6320	79.33% O ($sp^{2.37}$) + 20.67% H ($sp^{0.01}$)
double H-donor OH	O^1–H^a	0.6970	77.14% O ($sp^{2.81}$) + 22.86% H ($sp^{0.01}$)
double H-donor OH	O^1–H^b	0.6970	77.14% O ($sp^{2.81}$) + 22.86% H ($sp^{0.01}$)
H-donor-free OH	O^5–H^d	0.7507	75.15% O ($sp^{3.33}d^{0.01}$) + 24.85% H ($sp^{0.01}$)

Supplementary Table 13. Wiberg bond order and natural hybrid orbital of the single H-donor OH, double H-donor OH, and H-donor-free OH bonds in isomer **II** (S_4) for $(H_2O)_8$ calculated at the MP2/AVDZ level of theory (O, red; H, light gray).

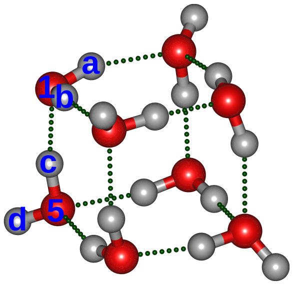

Type of OH group	bond	Wiberg bond order	Natural hybrid orbital
single H-donor OH	$O^5–H^c$	0.6316	79.33% O ($sp^{2.37}$) + 20.67% H ($sp^{0.01}$)
double H-donor OH	$O^1–H^a$	0.6978	77.17% O ($sp^{2.81}$) + 22.83% H ($sp^{0.01}$)
double H-donor OH	$O^1–H^b$	0.6963	77.12% O ($sp^{2.81}$) + 22.88% H ($sp^{0.01}$)
H-donor-free OH	$O^5–H^d$	0.7510	75.13% O ($sp^{3.33}d^{0.01}$) + 24.87% H ($sp^{0.01}$)

Supplementary Table 14. Wiberg bond order and natural hybrid orbital of the single H-donor OH, double H-donor OH, and H-donor-free OH bonds in isomer **III** (C_2) for $(H_2O)_8$ calculated at the MP2/AVDZ level of theory (O, red; H, light gray).

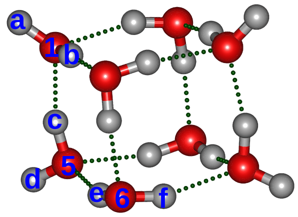

Type of OH group	bond	Wiberg bond order	Natural hybrid orbital
single H-donor OH	$O^1–H^b$	0.6135	79.91% O ($sp^{2.31}$) + 20.09% H ($sp^{0.01}$)
single H-donor OH	$O^5–H^c$	0.6659	78.26% O ($sp^{2.52}$) + 21.74% H ($sp^{0.01}$)
double H-donor OH	$O^6–H^f$	0.7106	76.62% O ($sp^{2.87}$) + 23.38% H ($sp^{0.01}$)
double H-donor OH	$O^6–H^e$	0.7132	76.52% O ($sp^{2.90}$) + 23.48% H ($sp^{0.01}$)
H-donor-free OH	$O^5–H^d$	0.7526	75.08% O ($sp^{3.30}d^{0.01}$) + 24.92% H ($sp^{0.01}$)
H-donor-free OH	$O^1–H^a$	0.7511	75.14% O ($sp^{3.37}d^{0.01}$) + 24.86% H ($sp^{0.01}$)

Supplementary Table 15. Wiberg bond order and natural hybrid orbital of the single H-donor OH, double H-donor OH, and H-donor-free OH bonds in isomer **IV** (C_2) for $(H_2O)_8$ calculated at the MP2/AVDZ level of theory (O, red; H, light gray).

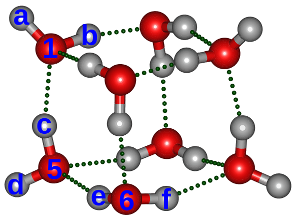

Type of OH group	bond	Wiberg bond order	Natural hybrid orbital
single H-donor OH	O^1–H^b	0.6136	79.91% O ($sp^{2.31}$) + 20.09% H ($sp^{0.01}$)
single H-donor OH	O^5–H^c	0.6661	78.26% O ($sp^{2.52}$) + 21.74% H ($sp^{0.01}$)
double H-donor OH	O^6–H^f	0.7132	76.52% O ($sp^{2.90}$) + 23.48% H ($sp^{0.01}$)
double H-donor OH	O^6–H^e	0.7106	76.62% O ($sp^{2.87}$) + 23.38% H ($sp^{0.01}$)
H-donor-free OH	O^5–H^d	0.7526	75.08% O ($sp^{3.30}d^{0.01}$) + 24.92% H ($sp^{0.01}$)
H-donor-free OH	O^1–H^a	0.7511	75.14% O ($sp^{3.37}d^{0.01}$) + 24.86% H ($sp^{0.01}$)

Supplementary Table 16. Wiberg bond order and natural hybrid orbital of the single H-donor OH, double H-donor OH, and H-donor-free OH bonds in isomer **V** (C_i) for $(H_2O)_8$ calculated at the MP2/AVDZ level of theory (O, red; H, light gray).

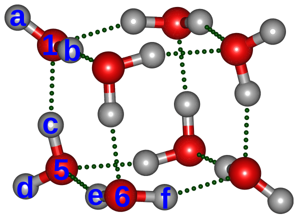

Type of OH group	bond	Wiberg bond order	Natural hybrid orbital
single H-donor OH	$O^1–H^b$	0.6133	79.91% O ($sp^{2.31}$) + 20.09% H ($sp^{0.01}$)
single H-donor OH	$O^5–H^c$	0.6659	78.26% O ($sp^{2.53}$) + 21.74% H ($sp^{0.01}$)
double H-donor OH	$O^6–H^f$	0.7113	76.59% O ($sp^{2.88}$) + 23.41% H ($sp^{0.01}$)
double H-donor OH	$O^6–H^e$	0.7139	76.50% O ($sp^{2.91}$) + 23.50% H ($sp^{0.01}$)
H-donor-free OH	$O^5–H^d$	0.7528	75.07% O ($sp^{3.31}d^{0.01}$) + 24.93% H ($sp^{0.01}$)
H-donor-free OH	$O^1–H^a$	0.7512	75.13% O ($sp^{3.37}d^{0.01}$) + 24.87% H ($sp^{0.01}$)

S3. Supplementary References

1. Buck, U., Ettischer, I., Melzer, M., Buch, V. & Sadlej, J. Structure and spectra of three-dimensional $(H_2O)_n$ clusters, $n = 8, 9, 10$. *Phys. Rev. Lett.* **80**, 2578-2581 (1998).
2. Gruenloh, C. J. *et al.* Infrared spectrum of a molecular ice cube: The S_4 and D_{2d} water octamers in benzene-(water)$_8$. *Science* **276**, 1678-1681 (1997).
