# Supplementary material for: Infrared spectroscopic study of hydrogen bonding topologies in the smallest ice cube
Source: Nat Commun. 2020 Oct 28;11:5449. doi: 10.1038/s41467-020-19226-6 (PMC7595032; doi:10.1038/s41467-020-19226-6)
Supplement: Supplementary file 3 — Description of Additional Supplementary Files [file 41467_2020_19226_MOESM3_ESM.docx]

Description of Additional Supplementary Files.

File Name: Supplementary Data 1
Description: Animation of vibrational normal modes of the three bands S, D, and F of the *D_2d_*, *S_4_*, *C_2_*, and *C_i_* isomers for the water octamer.

File Name: Supplementary Data 2

Description: Cartesian coordinates of isomers I-VI of (H2O)_8_ calculated at the MP2/aug-cc-pVDZ level of theory.
