# Supplementary material for: Infrared spectroscopic study of hydrogen bonding topologies in the smallest ice cube
Source: Nat Commun. 2020 Oct 28;11:5449. doi: 10.1038/s41467-020-19226-6 (PMC7595032; doi:10.1038/s41467-020-19226-6)
Supplement: Supplementary file 4 — Supplementary Data 1 [file 41467_2020_19226_MOESM4_ESM.pptx]

## Slide 1
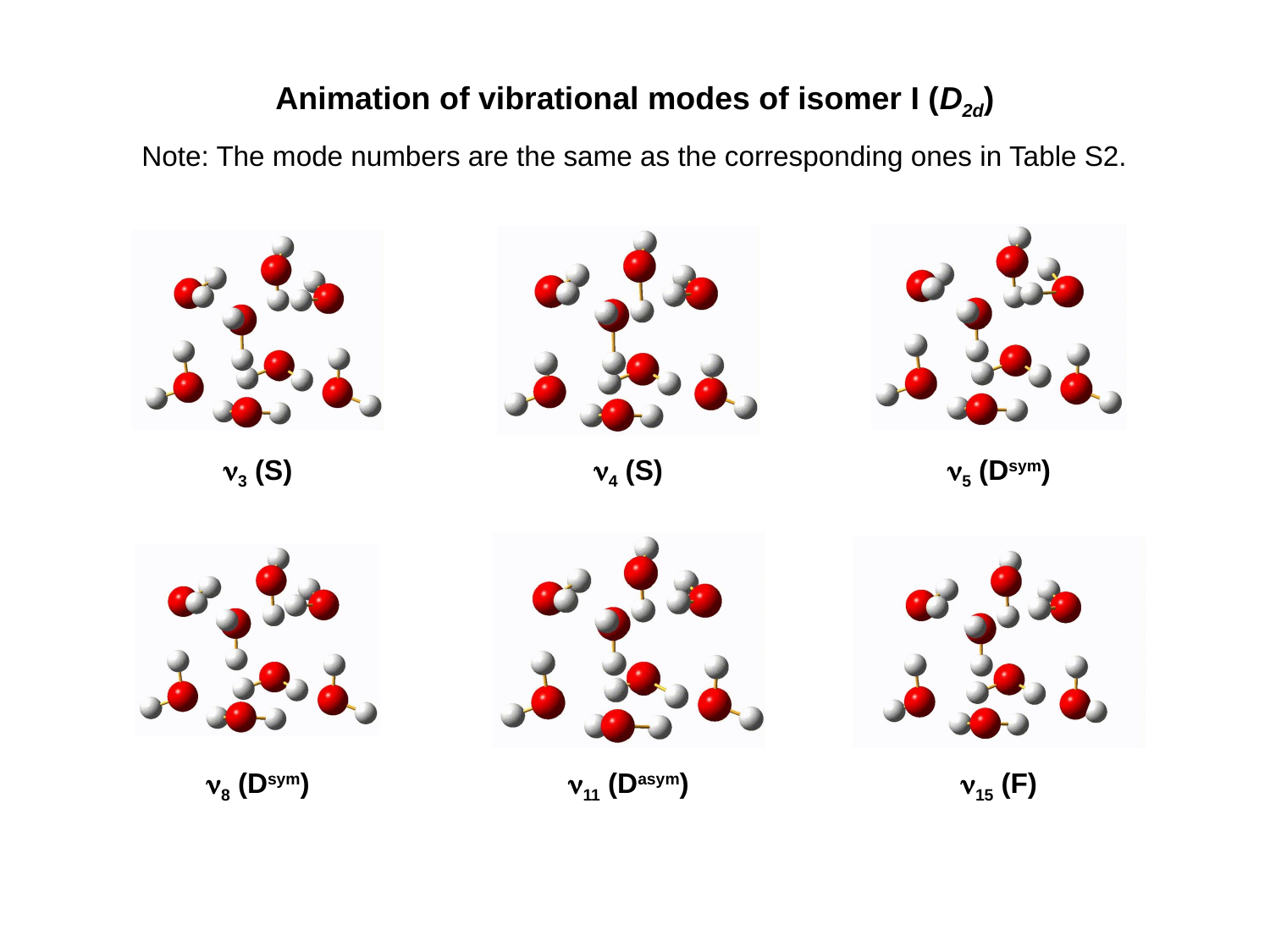

Animation of vibrational modes of isomer I (D2d)
Note: The mode numbers are the same as the corresponding ones in Table S2.
3 (S)
4 (S)
5 (Dsym)
8 (Dsym)
11 (Dasym)
15 (F)

## Slide 2
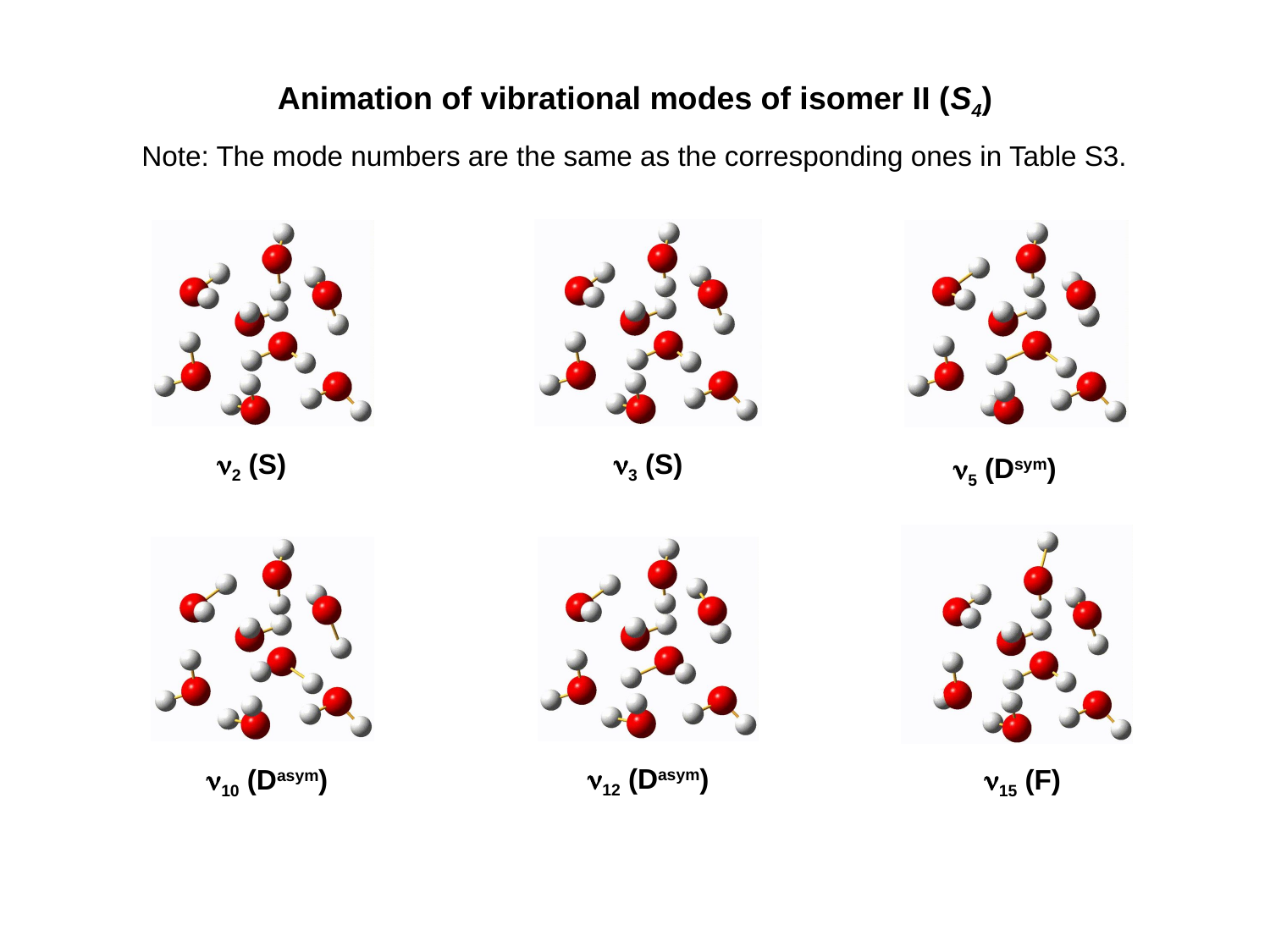

Animation of vibrational modes of isomer II (S4)
Note: The mode numbers are the same as the corresponding ones in Table S3.
3 (S)
2 (S)
5 (Dsym)
12 (Dasym)
10 (Dasym)
15 (F)

## Slide 3
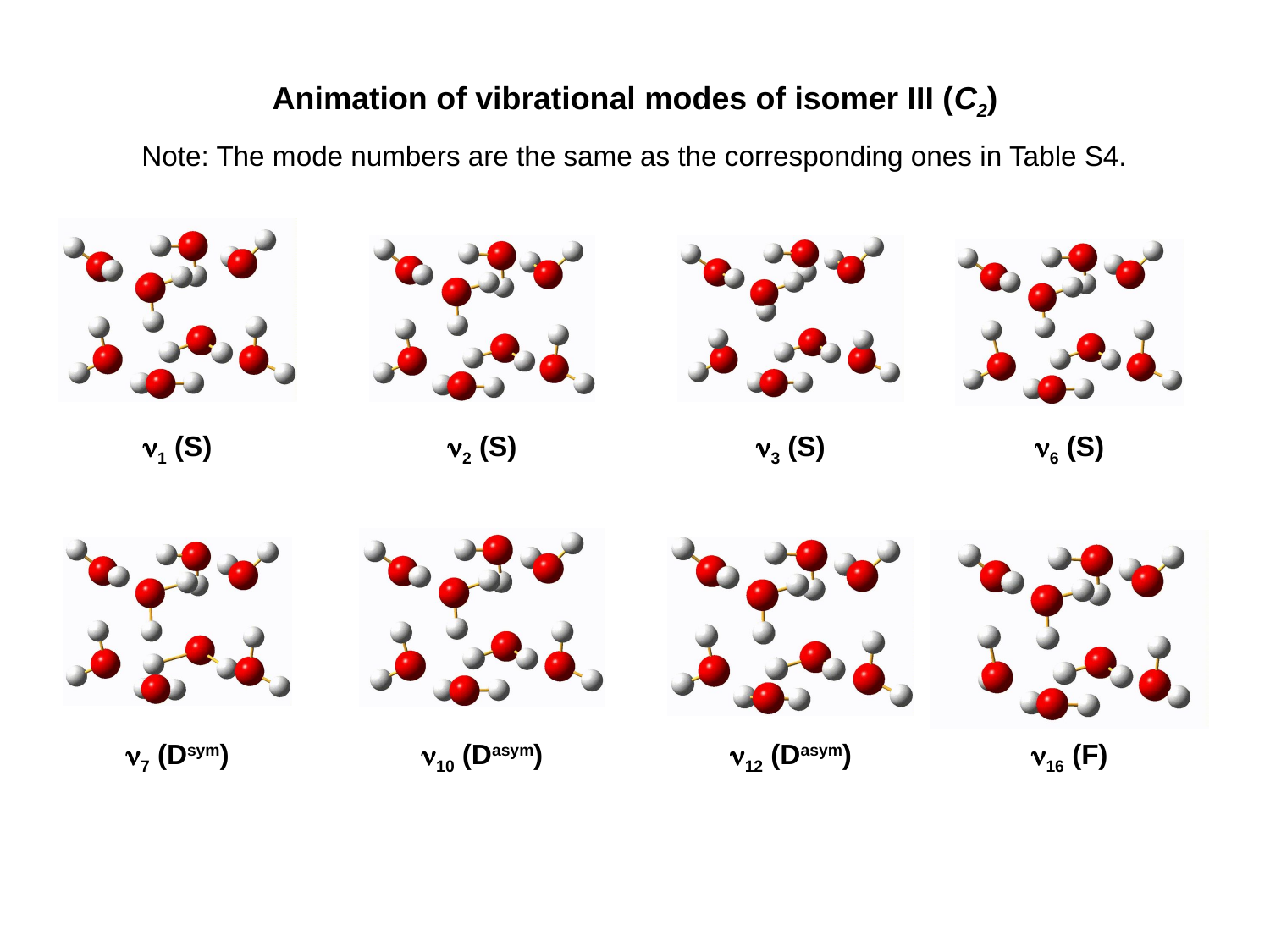

Animation of vibrational modes of isomer III (C2)
Note: The mode numbers are the same as the corresponding ones in Table S4.
1 (S)
2 (S)
3 (S)
6 (S)
7 (Dsym)
10 (Dasym)
12 (Dasym)
16 (F)

## Slide 4
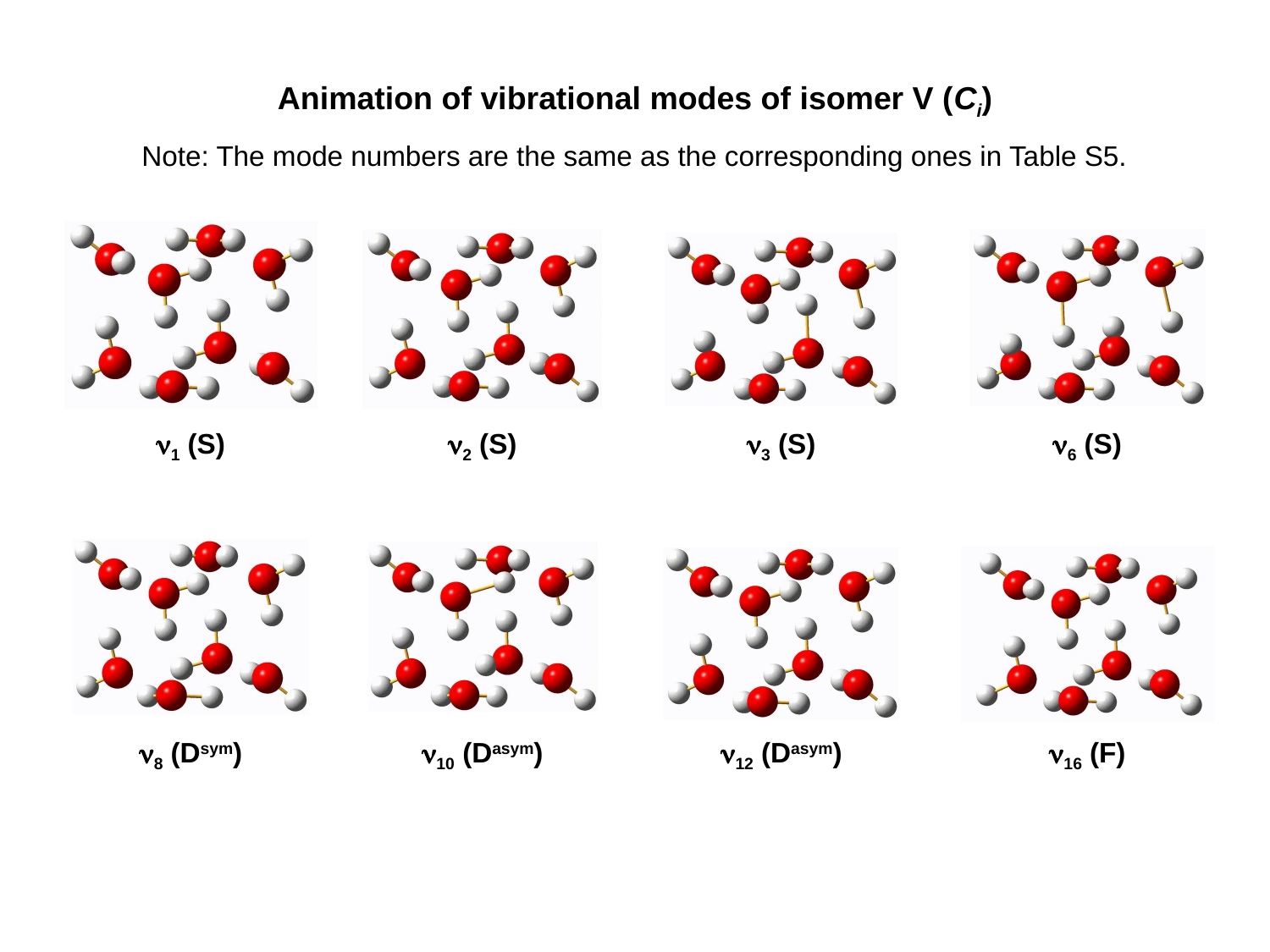

Animation of vibrational modes of isomer V (Ci)
Note: The mode numbers are the same as the corresponding ones in Table S5.
1 (S)
2 (S)
3 (S)
6 (S)
8 (Dsym)
10 (Dasym)
12 (Dasym)
16 (F)
